# Supplementary material for: Visual periodicity reveals distinct attentional signatures for face and non-face categories
Source: Cereb Cortex. 2024 Jun 16;34(6):bhae228. doi: 10.1093/cercor/bhae228 (PMC11180377; doi:10.1093/cercor/bhae228)
Supplement: Supplementary_Materials_bhae228 [file supplementary_materials_bhae228.docx]

# SUPPLEMENTAL MATERIALS


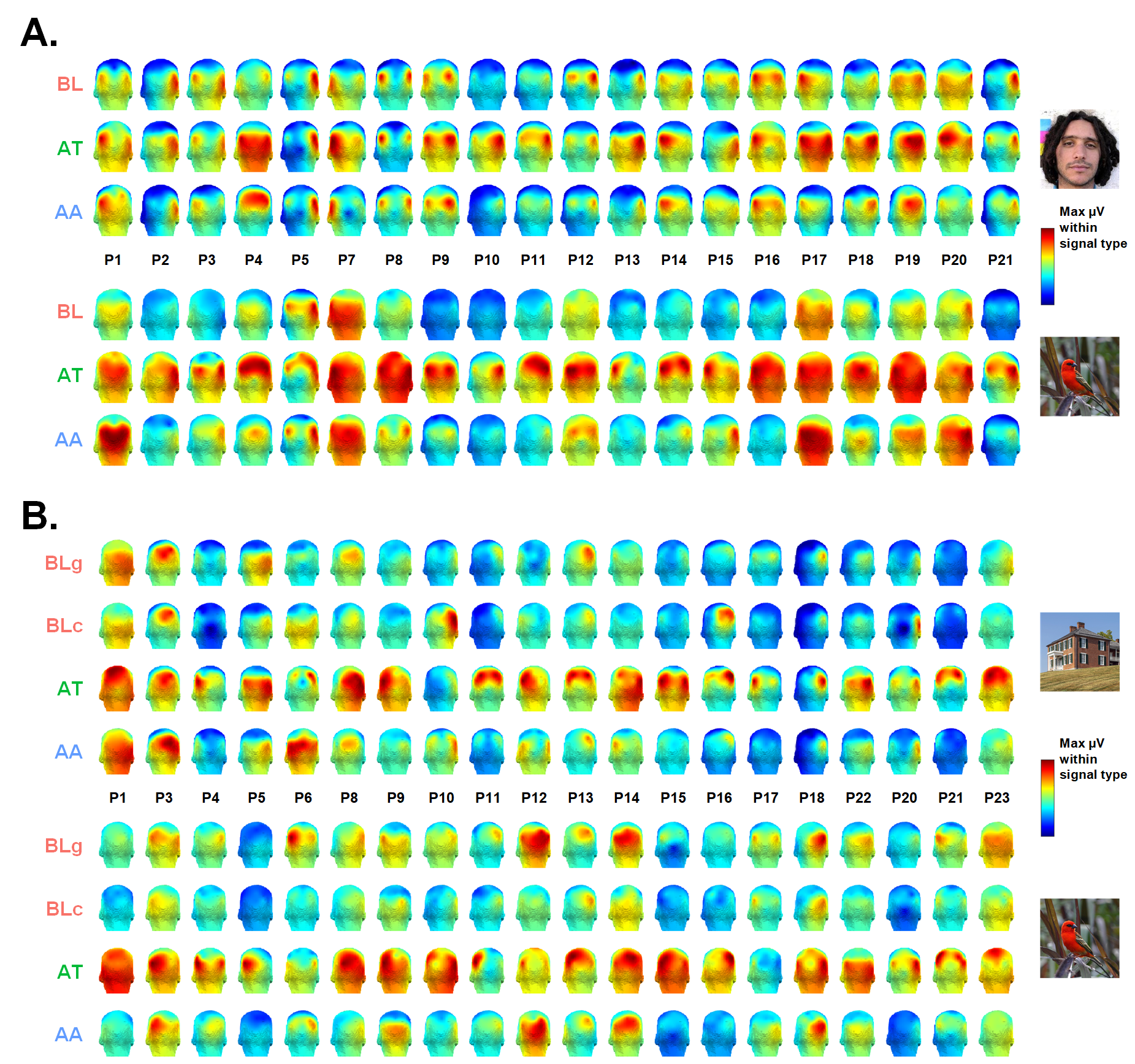


**Figure S1.** **A)** Individual participant scalp topographies for each category-selective response in each condition of Exp 1, shown as a function of attention condition (BL=baseline; AT = attend towards; AA = attend away). Amplitude ranges are fixed across condition, for each participant individually. **B)** The same for Exp 2 (BLg = baseline (cross); blG = baseline (guitar); AT = attend towards; AA = attend away).


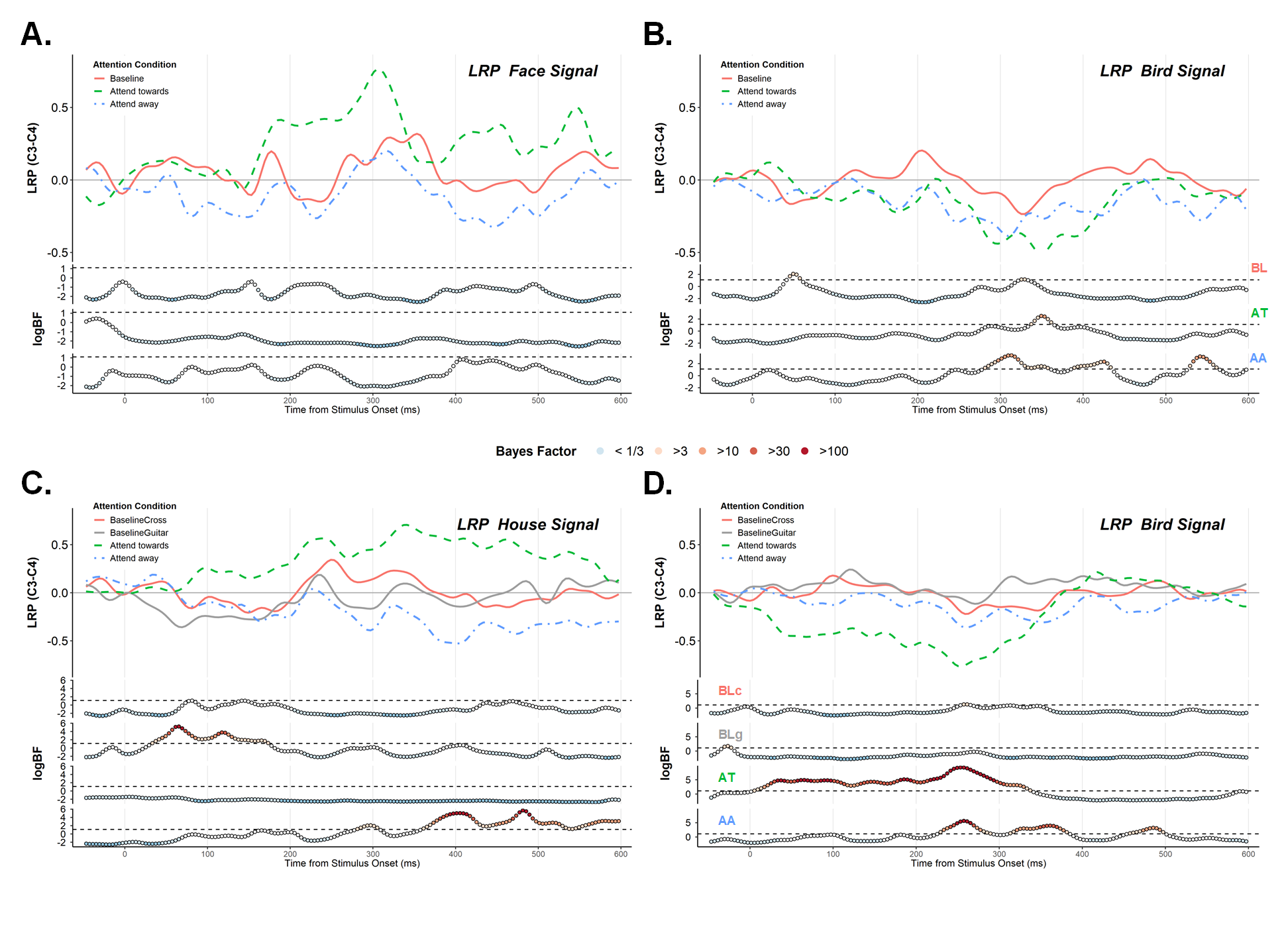


**Figure S2.** LRPs (electrode C3-C4) calculated using each participant’s baseline-corrected conditional mean epoch locked to the onset of **A)** faces in Exp 1, **B)** birds in Exp 1, **C)** houses in Exp 2, and **D)** birds in Exp 2. Attentional conditions are plotted separately; lower panels show log Bayes Factors testing values < 0 (i.e., a classic negative deflection reflecting motor preparation). We were specifically interested in whether the ‘Attend Towards’ condition in which participants pressed a button with their right hand each time the critical category appeared on the screen would drive a pronounced LRP. There was no evidence of such a pattern in 3 out of 4 cases.


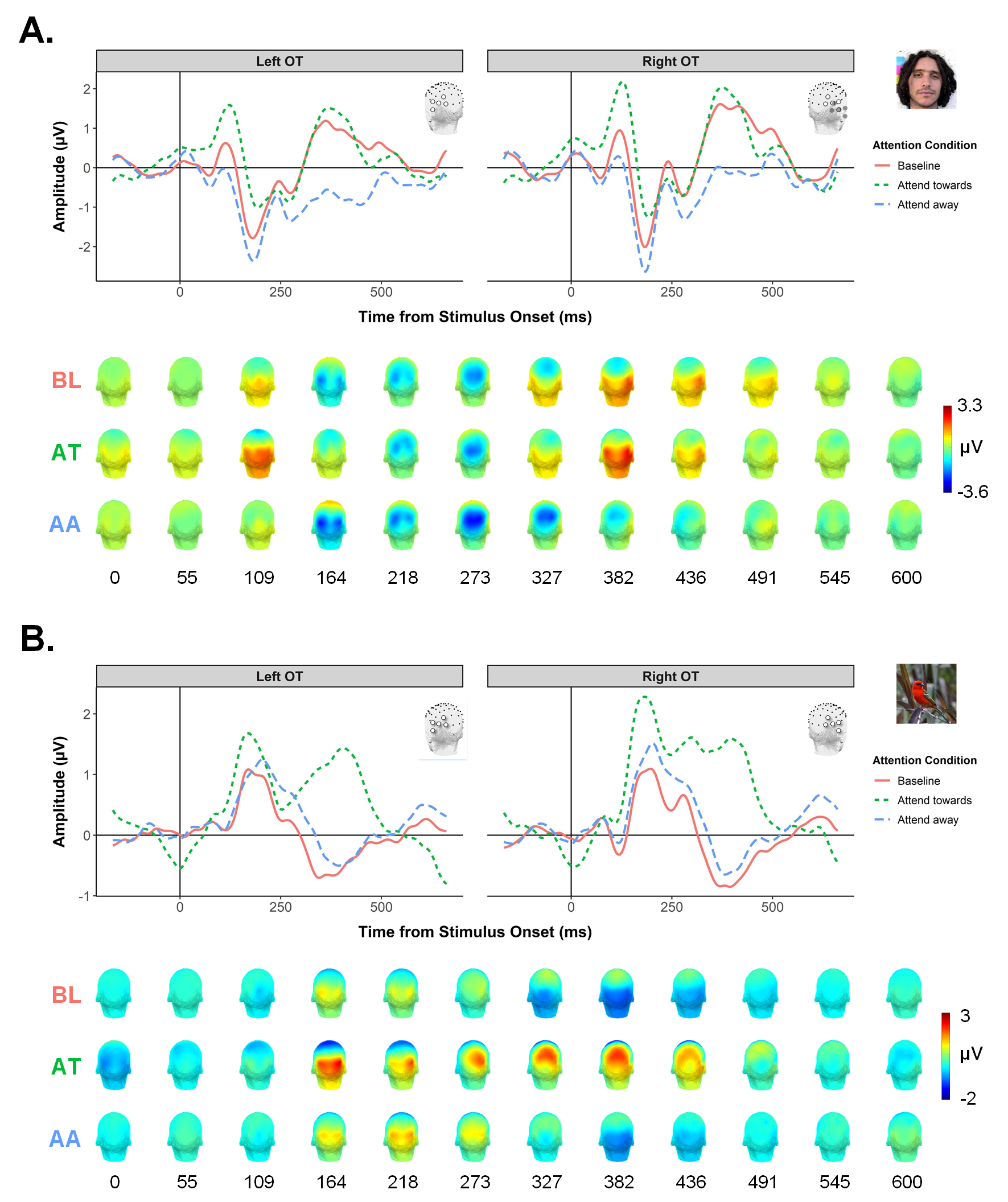


**Figure S3.** The face-selective (A) and bird-selective responses (B) for Exp 1 shown in the time-domain. Waveforms for each attentional condition are plotted separately for the left and right occipitotemporal ROIs. Group-mean scalp topographies for each condition are shown below each category-selective response for a range of timepoints between 0 and 600ms (BL=baseline; AT = attend towards; AA = attend away).


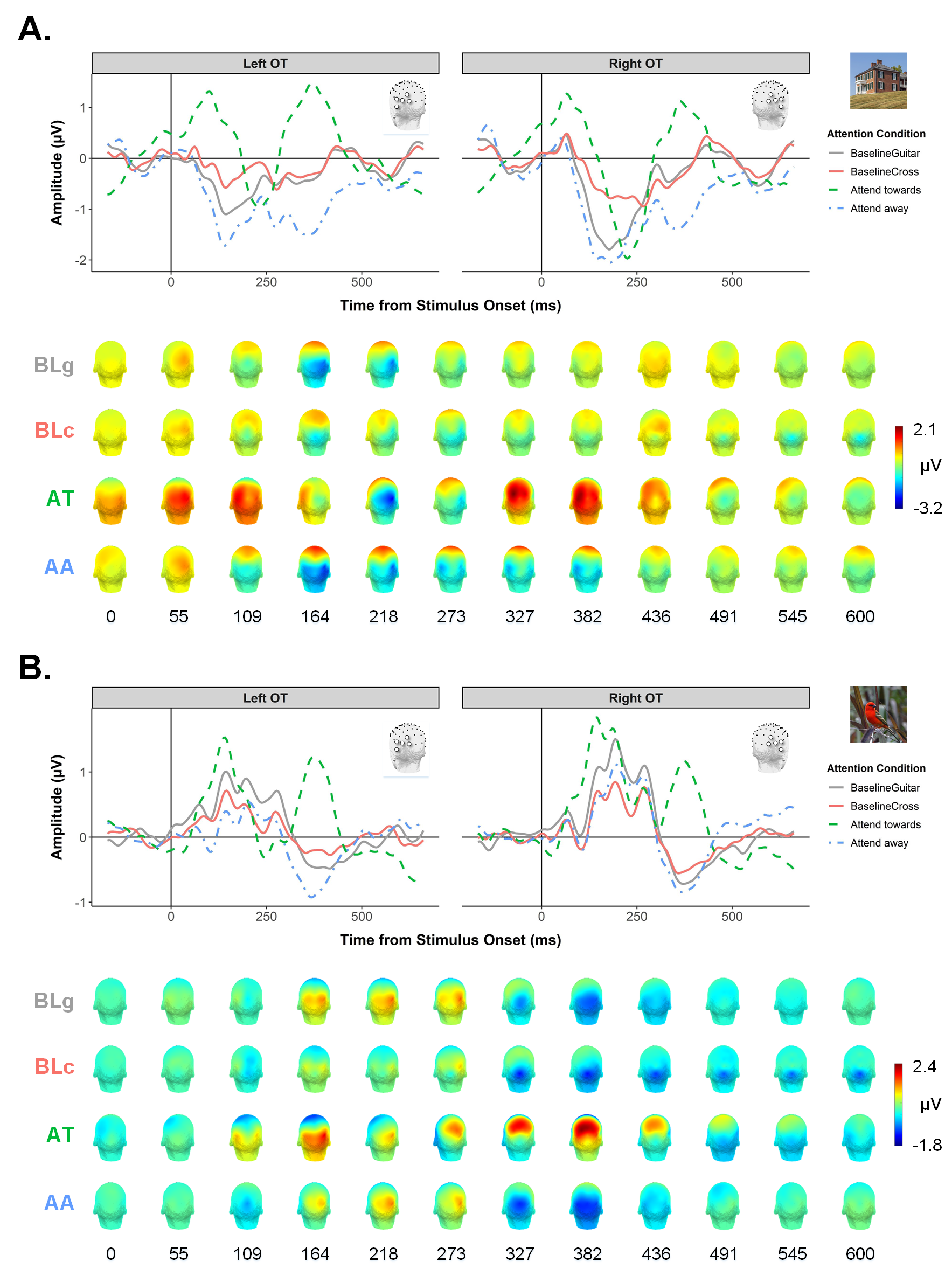


**Figure S4.** The house-selective response (A) and bird-selective response (B) for Exp 2 shown in the time-domain. Waveforms for each attentional condition are plotted separately for the left and right occipitotemporal ROIs. Group-mean scalp topographies for each condition are shown below each category-selective response for a range of timepoints between 0 and 600ms (BLg = baseline guitar; BLc = baseline cross; AT = attend towards; AA = attend away).


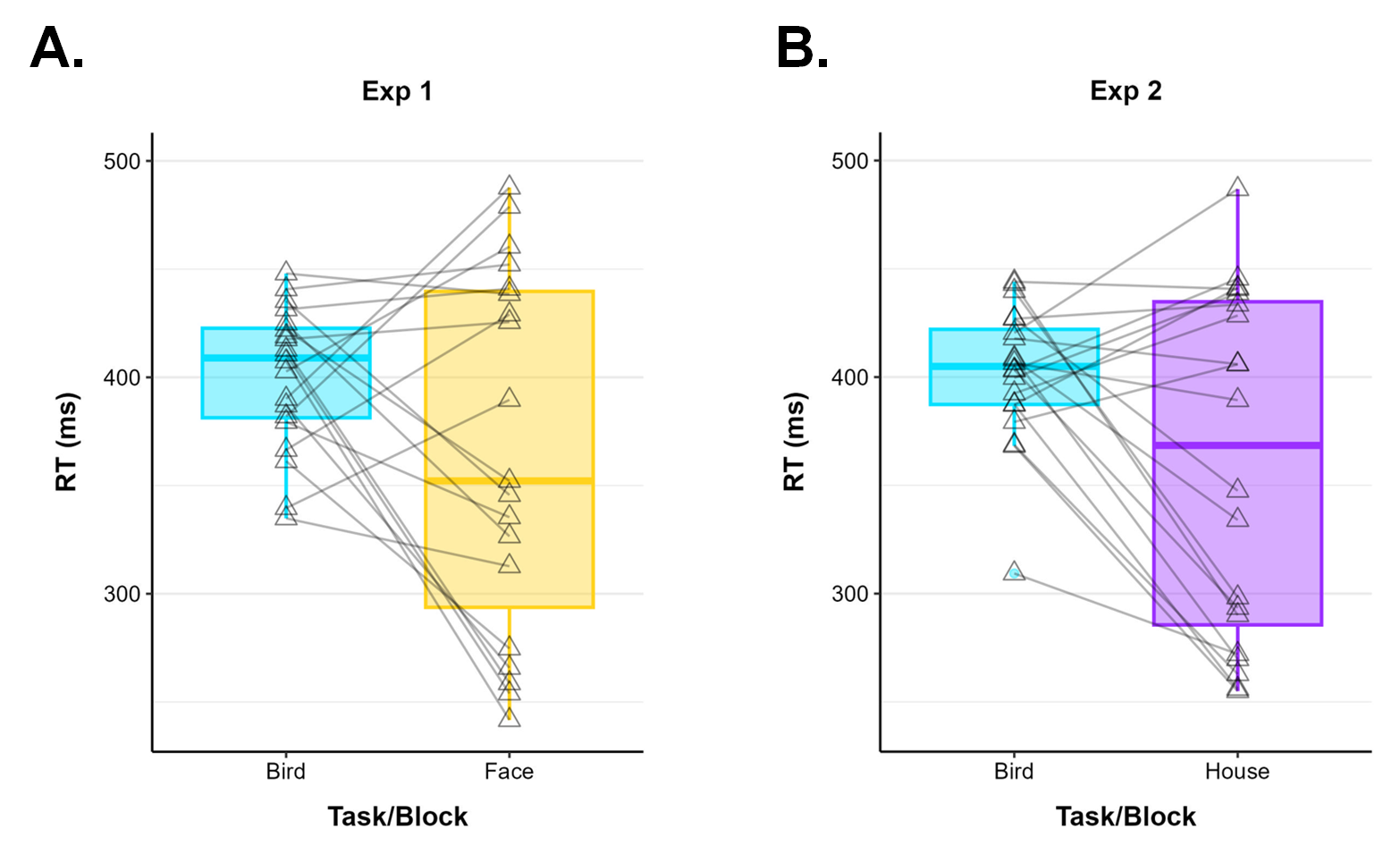


**Figure S5.** Response time (RT) data indexing for participants’ detection of the critical categories in (A) Exp 1 (birds/faces) and (B) Exp 2 (birds/houses). Overlaid triangles are individual participants; note that different participant samples were used across the two experiments. In both cases, mean RT was higher for bird-detection responses than for detection responses to the alternate category (Exp 1: *t*(19) = 2.01, *p* = 0.059 ; Exp 2: *t*(19) = 2.53, *p* = 0.021). RTs for both faces and houses also tended to be much more variable than RTs for birds.
